# Health-system-adapted data envelopment analysis for decision-making in universal health coverage

**DOI:** 10.2471/BLT.17.191817

**Published:** 2018-04-23

**Authors:** Mark G Shrime, Swagoto Mukhopadhyay, Blake C Alkire

**Affiliations:** a Program in Global Surgery and Social Change, Harvard Medical School, 641 Huntington Ave #411, Boston, Massachusetts, 02115, United States of America (USA).; b Department of Surgery, University of Connecticut, Farmington, USA.

## Abstract

**Objective:**

To develop and test a method that allows an objective assessment of the value of any health policy in multiple domains.

**Methods:**

We developed a method to assist decision-makers with constrained resources and insufficient knowledge about a society’s preferences to choose between policies with unequal, and at times opposing, effects on multiple outcomes. Our method extends standard data envelopment analysis to address the realities of health policy, such as multiple and adverse outcomes and a lack of information about the population’s preferences over those outcomes. We made four modifications to the standard analysis: (i) treating the policy itself as the object of analysis, (ii) allowing the method to produce a rank-ordering of policies; (iii) allowing any outcome to serve as both an output and input; and (iv) allowing variable return to scale. We tested the method against three previously published analyses of health policies in low-income settings.

**Results:**

When applied to previous analyses, our new method performed better than traditional cost–effectiveness analysis and standard data envelopment analysis. The adapted analysis could identify the most efficient policy interventions from among any set of evaluated policies and was able to provide a rank ordering of all interventions.

**Conclusion:**

Health-system-adapted data envelopment analysis allows any quantifiable attribute or determinant of health to be included in a calculation. It is easy to perform and, in the absence of evidence about a society’s preferences among multiple policy outcomes, can provide a comprehensive method for health-policy decision-making in the era of sustainable development.

## Résumé

**Objectif:**

Développer et tester une méthode permettant d'évaluer objectivement la valeur de toute politique sanitaire dans de multiples domaines.

**Méthodes:**

Nous avons développé une méthode pour aider les décideurs qui possèdent des ressources limitées et des connaissances insuffisantes concernant les préférences d'une société à choisir entre des politiques ayant des effets inégaux, et parfois opposés, sur de multiples résultats. Notre méthode élargit l'analyse standard de l'enveloppement de données pour tenir compte des réalités d'une politique sanitaire, et notamment de résultats multiples et négatifs et d'un manque d'informations concernant les préférences d'une population à l'égard de ces résultats. Nous avons apporté quatre modifications à l'analyse standard: (i) nous avons pris la politique elle-même comme objet d'analyse; (ii) nous avons fait en sorte que la méthode permette de classer les politiques; (iii) nous avons veillé à ce que chaque résultat serve à la fois d'entrée et de sortie; et (iv) nous avons prévu un rendement d'échelle variable. Nous avons testé la méthode par rapport à trois analyses précédemment publiées de politiques sanitaires dans des pays à faibles revenus.

**Résultats:**

Appliquée aux précédentes analyses, notre nouvelle méthode a donné de meilleurs résultats qu'une traditionnelle analyse coût-efficacité et qu'une analyse standard de l'enveloppement de données. Cette analyse adaptée a permis de repérer les interventions les plus efficaces parmi un ensemble de politiques évaluées et d'établir un classement de toutes les interventions.

**Conclusion:**

L'analyse de l'enveloppement de données adaptée au système de santé permet d'inclure dans un calcul toute caractéristique ou tout déterminant quantifiable de la santé. Cette analyse est facile à réaliser et, en l'absence de données concernant les préférences d'une société parmi plusieurs résultats de politique, elle fournit une méthode complète pour la prise de décision en matière de politique sanitaire à l'ère du développement durable.****

## Resumen

**Objetivo:**

Desarrollar y probar un método que permita una evaluación objetiva del valor de cualquier política de salud en múltiples dominios.

**Métodos:**

Se desarrolló un método para ayudar a los responsables de la toma de decisiones con recursos limitados y conocimientos insuficientes sobre las preferencias de una sociedad para elegir entre políticas de efectos desiguales, y en ocasiones opuestos, en resultados múltiples. El método amplía el análisis envolvente de datos estándar para abordar las realidades de la política de salud, como los resultados múltiples y adversos y la falta de información sobre las preferencias de la población con respecto a dichos resultados. Se realizaron cuatro modificaciones al análisis estándar: (i) tratar la política en sí misma como el objeto de análisis; (ii) permitir que el método produzca un orden jerárquico de las políticas; (iii) permitir que cualquier resultado sirva como entrada y salida; y (iv) permitir el rendimiento variable a escala. Se probó el método en comparación con tres análisis publicados anteriormente de políticas de salud en entornos de bajos ingresos.

**Resultados:**

Cuando se aplicó a análisis previos, el nuevo método funcionó mejor que el análisis tradicional de coste y efectividad y que el análisis estándar envolvente de datos. El análisis adaptado identificó las intervenciones de políticas más eficaces de entre un conjunto de políticas evaluadas y proporcionó un orden jerárquico de todas las intervenciones.

**Conclusión:**

El análisis envolvente de datos adaptado al sistema de salud permite incluir cualquier atributo o determinante de salud cuantificable en un cálculo. Es fácil de realizar y, a falta de evidencia sobre las preferencias de una sociedad entre múltiples resultados de una política, proporciona un método integral para la toma de decisiones sobre políticas de salud en la era del desarrollo sostenible.

## ملخص

**الغرض:**

تطوير واختبار طريقة تتيح إجراء تقييم موضوعي لقيمة أي سياسة صحية في العديد من المجالات.

**الطريقة:**

قمنا بتطوير أسلوب لمساعدة صناع القرار المعتمدين على موارد محدودة ومعرفة غير كافية حول أفضليات المجتمع للاختيار ما بين السياسات ذات الآثار غير المتكافئة – والتي أحيانًا ما تكون متعارضة – على المحصلات المتعددة. ويعمل الأسلوب الذي نقدمه على مد نطاق التحليل القياسي لتغطية البيانات بحيث يتعامل مع وقائع السياسة الصحية، مثل تعدد المحصلات الناتجة ونشوء محصلات سلبية، ونقل المعلومات حول أفضليات الشرائح السكانية فيما يتعلق بتلك المحصلات. وقد أجرينا أربعة تعديلات على التحليل القياسي تتمثل فيما يلي: علاج السياسة ذاتها بصفتها هدف للتحليل، و(ب) السماح للأسلوب بإنتاج تصنيف تراتبي للسياسات، و(جـ) السماح باعتبار محصلة بمثابة ناتج ومعطيات في نفس الوقت، و(د) السماح باحتساب عوائد متغيرة الحجم. وقد اختبرنا الأسلوب في ضوء ثلاثة تحليلات سبق نشرها في الماضي للسياسات الصحية في البيئات التي تسودها مستويات الدخل المنخفضة.

**النتائج:**

عند تطبيق الأسلوب المقترح من جانبنا على التحليلات السابقة، فقد حقق هذا الأداء الأسلوب الجديد أداءً أفضل من التحليل التقليدي لفعالية التكلفة والتحليل القياسي لتغطية البيانات. وأمكن للتحليل المعدّل أن يحدد التدخلات الأكثر فعالية في السياسة من بين أي مجموعة من السياسات التي خضعت للتقييم، كما أمكنه تقديم تصنيف تراتبي لجميع التدخلات.

**الاستنتاج:**

يتيح تحليل تغطية البيانات المعدَّل حسب النظم الصحية تضمين أي خاصية أو محدِّد للصحة قابل للقياس الكمّي في عملية حسابية. ومن السهل إجراء هذا التحليل، كما يمكنه في غياب الأدلة حول الأفضليات السائدة في أحد المجتمعات أن يقدم أسلوبًا شاملاً لعملية اتخاذ القرار بشأن السياسات الصحية في عصر التنمية المستدامة.

## 摘要

**目的:**

开发并测试一种可客观评估多领域任意卫生政策价值的方法。

**方法:**

我们开发了一种方法，在资源有限且对社会偏好缺乏足够了解的情况下，协助决策者在对多个结果有不平等（时而相反）效果的政策间做出选择。我们的方法拓展了标准的数据包络分析以解决卫生政策的现实问题，例如产生多种相反结果、缺乏针对这些结果的群体偏好信息。我们对标准分析作了 4 次修改：(i) 将政策本身视为分析对象；(ii) 可采用该方法为政策排序；(iii) 任何结果既可作为输出信息,又可作为输入信息；和 (iv) 可测量浮动回报。我们采用之前发布的三种低收入国家卫生政策分析来测试该方法。

**结果:**

我们的新方法用于先前分析时，比传统成本效益分析和标准数据包络分析的效果更好。调整后的分析可从任何一组已评估的政策中确定最有效的政策干预措施，并能将所有干预措施排序。

**结论:**

适用于卫生系统的数据包络分析使任何卫生的度量类属性或决定因素均可纳入计算。该分析易于进行，在可持续发展时代，在缺乏社会对多政策结果偏好证据的条件下，它能为卫生政策决策制定提供一种综合方法。****

## Резюме

**Цель:**

Разработать и протестировать метод, который позволил бы объективно оценивать значение любой политики в различных сферах деятельности в области здравоохранения.

**Методы:**

Авторы разработали метод, содействующий принятию решений при выборе между политиками с неравными и порой противоположными последствиями уполномоченными лицами в условиях ограниченных ресурсов и недостаточных знаний о предпочтениях общества. Этот метод позволяет расширить применение стандартного анализа охвата данных для учета реалий политики здравоохранения, таких как многочисленные и неблагоприятные последствия, а также отсутствие информации об отношении населения к этим последствиям. Авторы внесли четыре изменения в стандартный анализ: (i) рассмотрение самой политики как объекта анализа; (ii) включение в метод возможности проведения ранжирования политики; (iii) включение возможности для любого последствия служить в качестве как исходных данных, так и результатов; (iv) включение возможности переменного эффекта масштабирования. Авторы протестировали этот метод на основе трех ранее опубликованных анализов политики здравоохранения в условиях низкого уровня дохода.

**Результаты:**

При применении на основе предыдущего анализа новый метод продемонстрировал лучшие результаты, чем традиционный анализ экономической эффективности и стандартный анализ охвата данных. Адаптированный анализ способен определить наиболее эффективные политические меры среди любого набора оцениваемых стратегий и может обеспечить ранжирование всех мер.

**Вывод:**

Анализ охвата данных, адаптированный к системе здравоохранения, позволяет включать в расчет любой количественный атрибут или детерминант здоровья. Такой анализ прост в осуществлении и при отсутствии информации об отношении общества к нескольким последствиям политики может обеспечить комплексный метод принятия решений в области здравоохранения в эпоху устойчивого развития.

## Introduction

In 2015, the United Nations adopted 17 sustainable development goals, reflecting a commitment to end poverty in all forms by 2030. Among the targets of the third goal is the establishment of universal health coverage (UHC),^1^ ensuring “all people and communities can use the promotive, preventive, curative, rehabilitative and palliative health services they need, of sufficient quality to be effective, while also ensuring that the use of these services does not expose the user to financial hardship”.^2^ Achieving this requires countries to expand the number of health conditions covered, improve the quality of services, increase the number of people covered and provide protection against financial risk.^3^

Health policy decision-making is complicated, however, by the fact that no health policy can improve coverage, equity, quality and financial risk protection simultaneously and to the same degree.^4^ This forces policy-makers to confront challenging resource-allocation questions: Is it more important for society to cover more people, treat more conditions, improve equity or increase financial protection? Ideally, choosing among different policies (Box 1) requires knowledge about the population’s preferences, knowledge which may not exist.

Box 1 **Three hypothetical policy interventions that illustrate trade-offs in health policy decision-making**

**Policy A**

Policy characteristics:

- Cost US$ 175 000
- 200 deaths averted
- 40 cases of catastrophic health expenditure created
- Mildly favours richer patients

This policy improves health the most, but is mildly regressive and creates catastrophic medical expenditure for patients

**Policy B**

Policy characteristics:

- Cost US$ 150 000
- 40 deaths averted
- 20 cases of catastrophic health expenditure averted
- Mildly favours poorer patients

This policy is less regressive than Policy A and provides financial risk protection, (i.e. negative cases of catastrophic expenditure created) but delivers the least health benefit.

**Policy C**

Policy characteristics:

- Cost US$ 200 000
- 80 deaths averted
- 60 cases of catastrophic health expenditure created
- Strongly favours poorer patients

This policy is the most equitable of the three and provides a moderate amount of health improvement, but creates the most financial catastrophe and is the most expensive.

**Choosing among these policies**

Ideally, choosing among the three would require knowledge about the target population’s preference weights across health, financial risk protection, equity and cost. In the absence of such knowledge, balancing the competing outcomes is difficult, and is the subject of the method presented here.

US$: United States dollars.

Analytical models such as extended cost‒effectiveness analyses can make the health, financial and equity effects of policies explicit.^4^^–^^8^ The newest recommendations of the Second Panel on Cost–Effectiveness in Health and Medicine advocate including an impact inventory of the non-health outcomes of medical interventions, such as economic productivity.^9^^,^^10^ However, other than simply reporting multiple outcomes, no method exists for decision-making that balances these many, and sometimes conflicting, domains.

This paper describes the development of a method for health policy decision-making in the absence of knowledge about a society’s preferences, with modifications for dealing with undesired outcomes. The method is an extension of standard data envelopment analysis, adapted for health policymaking; it combines the costs of health policies with their effects on multiple disparate domains into a single rank-ordering. We evaluated the method by applying it to the findings of three previous extended cost–effectiveness analyses.

## Methods

### Measuring value in health

The literature of cost–effectiveness research,^11^ and, more recently, of value-based health care^12^ has defined value as:
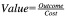
(1)Although theoretically attractive, operationalizing this ratio is difficult when there are multiple inputs and outputs.

To illustrate the concept of preference weighting we can consider two health-care policies: (i) training community health workers, which costs United States dollars (US$) 10 000, requires 10 faculty, averts 500 disability-adjusted life-years, and prevents 10 instances of catastrophic expenditure annually; or (ii) training specialists, which costs US$ 100 000, requires 20 faculty, and averts 600 disability-adjusted life-years and 12 instances of catastrophic expenditure annually. Cost‒effectiveness analysis looks only at costs and health benefits. The first policy costs US$ 20 per disability-adjusted life-years averted, while the second policy carries an incremental cost‒effectiveness ratio^11^ of US$ 900 disability-adjusted life-years averted. Decision-making is straightforward: if these ratios are less than society’s willingness to pay, the policy is deemed cost–effective.

This betrays an underlying assumption, not consistent with reality: that health effects and costs are sufficient metrics for decision-making. Patients, for example, may choose health care based on other factors such as affordability, satisfaction, distance or time. How people judge these trade-offs (that is, their underlying preference structure) is unknown. Furthermore, this preference structure is likely to vary across patients, be difficult to assess and not predicted by patients’ demographics.^13^ Patient-centred policy, then, must account for the fact that health effects and costs are valued against other inputs and outcomes. At the same time, laborious assessments of preference structures for every policy decision are impossible. 

Equation (1) can be extended to encompass more fully the examples above, including the domains of personnel and financial catastrophe, in addition to health and cost:
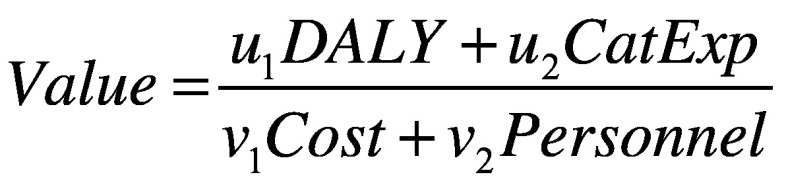
(2)The preference weight coefficients (*u* and *v*) formalize the trade-offs inherent in decision-making; that is, how important health and costs are relative to other outputs and inputs (cost‒effectiveness assumes *u*_2_ and *v*_2_ are zero).

Instead of attempting to determine the population’s values for *u* and *v*, our proposed method sets the preference weights as unknown and solves for them instead. To do so, it must impose two constraints: (i) the value of any policy must remain between 0 and 1 (inclusive); and (ii) *u* and *v* must take some positive value. With these constraints in place, the analysis finds solutions for *u* and *v* such that the value of each policy is as high as possible, while the values for all other potential policies, using these same preference weights, meet the constraints set above. This allows each policy to be judged on its own merits.

#### Data envelopment analysis

To calculate value of a policy without specifying the relative importance of inputs and outputs, the analysis instead allows each policy to set its own preference weights. Mathematically, we start with the first policy, *p_o_*, out of a set of *K* total policies. *p_o_* will use some amount of input (*x*) and produce some amount of output (*y*). The value of *p_o_*, which we call *θ_o_*, is a generalization of Equation (2): 
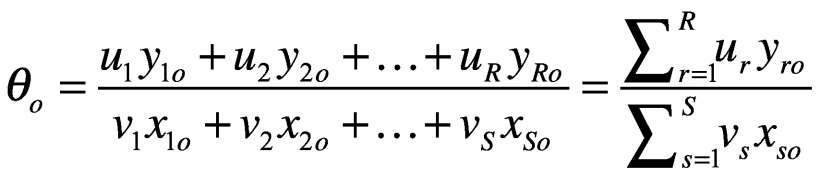
(3)Importantly, inputs and outputs are treated as having no units of measurement. That is, inputs could include square feet of hospital space, numbers of nurses and costs of the policy, while outputs could include deaths averted, impacts on a country’s gross domestic product and measures of equity.

The constraints imposed above make this a linear optimization problem in which *θ_o_* is maximized such that all efficiencies for all *K* policies are at most 1, and no policy is allowed to put zero weight on any input or output:
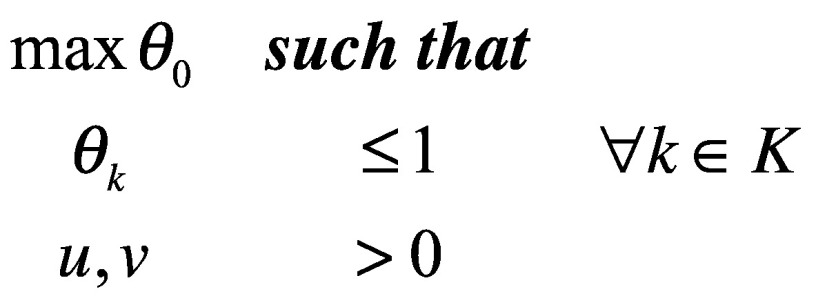
(4)A value of 1 suggests that no other policy is producing more outputs for a given set of inputs than *p*_0_. A value < 1 implies that *p*_o_ could do better (that is, other policies would convert its inputs into more outputs more efficiently).

Note that because the efficiency for each policy is calculated using the optimization, the relative importance weights, *u* and *v*, are recalculated for each policy. As a result, any inefficient policy can no longer be blamed on some external imposition of weights. Unfortunately, because each policy sets its own weights, it is conceivable, and in fact likely, for many to appear efficient, leaving the policy-maker with little guidance.

### Modifications for health policy

Four additional modifications are necessary to adapt data envelopment analysis to health policy applications. The first modification, already done above, is to treat the policy itself as the object of analysis, as opposed to any policy-maker, hospital or provider. This can be done because policies have direct consequences on the population’s health, financial well-being and equity (that is, they have direct outputs). Doing so requires decisions about cost, workforce training, infrastructure development and other inputs.

The second modification addresses the problem posed by multiple efficient policies. In real-world applications of Equation (4), many policies end up having a value of 1 (the maximum), which does not help the policy-maker. To produce a rank-ordering of policies, the first constraint in Equation (4) must be relaxed: in this so-called superefficiency analysis *θ_o_* is allowed to be larger than 1, while values for every other policy remain constrained.
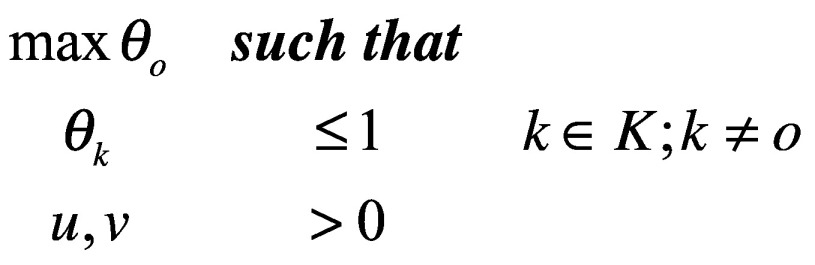
(5)*θ_o_* is calculated for the first policy, subject to the constraint that the value of all other policies remain between 0 and 1. This *θ_1_* is recorded, and the cycle repeats itself for the second policy. When *θ_2_* is calculated it is allowed to be larger than 1, but in that calculation, *θ_1_* is constrained. Once this calculation is done, *θ_2_* is recorded, and Equation (5) is repeated for the third decision-making unit, and so on.

This relaxation of constraints begins to produce rank orderings of health policies. However, a third modification is required. Equations (4) and (5) assume a constant return to scale, that is, that each additional unit of inputs (e.g. costs, personnel), will produce exactly the same unit of outputs as the one before. This is unlikely to be true in health. Policies that put a single surgeon in a previously unstaffed hospital, for example, are likely to return a significantly larger health benefit than those adding a second surgeon to a hospital that already has one. Allowing variable return to scale requires some added calculation, which has been developed elsewhere.^14^ Infeasibility is contravened by the Cook modification.^15^

One final modification is necessary to apply data envelopment analysis to health policy. In manufacturing, from which the method is derived,^16^ a producer cannot produce negative numbers of a product. In rare cases of negative outputs, the standard practice is to scale manufacturers’ outputs such that negative production no longer happens. That is, if a factory produces, 20 units of a product, 20 units of that product are simply added to the output of all factories, such that the negatively-producing manufacturer now produces 0, and every other manufacturer produces 20 more than previously. Although this may be mathematically justified, the translation to health is tenuous. For example, some policies can improve health, but worsen catastrophic out-of-pocket expenses for patients, thereby producing negative financial risk protection. Linear scaling would imply that such policies no longer produce any impoverishment, but that all other policies arbitrarily now provide even more protection against impoverishment. The probability that patients would find these two scenarios equivalent is low, making such scaling unhelpful to a decision-maker. A health-policy-adapted framework must take this into account.

We therefore allowed any outcome to serve as both an output and an input. For example, in cases of negative financial risk protection (that is, increased catastrophic expense) the additional financial risk produced by a policy is counted as a cost (or input) to the analysis. When catastrophic expense is prevented, the financial risk protection is counted as an output of the analysis. This modification penalizes policies with negative outcomes by increasing the size of the denominator in Equation (1), thereby decreasing that policy’s efficiency.

### Data sources and analysis

We tested our health-adapted superefficiency data envelopment analysis method by applying it to data from three previously published extended cost‒effectiveness analyses of policy inteventions.^4^^,^^5^^,^^8^ The first example was an analysis of policies to increase access to surgery in Ethiopia in terms of the cost, health benefits and effects on financial risk protection (Table 1). The second example was a synthesis of different preventive and curative health interventions from several analyses, reporting the cost, health benefits and financial risk protection of the interventions (Table 2). The third example looked at both government policy interventions and nongovernmental platforms for improving access to surgical cancer care in Uganda in terms of cost, deaths averted, cases of impoverishment averted and equity (Table 3).

**Table 1:** Extended cost–effectiveness analysis of policy interventions to increase access to surgery in rural Ethiopia

Intervention	Cost, US$	No. of deaths averted	No. of cases of impoverishment averted
Universal public finance	945 313	22.99	360.71
Universal public finance + vouchers	5 516 092	58.64	2 646.68
Task-shifting	401 491	252.55	−578.43
Universal public finance + task-shifting	2 354 435	289.12	−231.17
Universal public finance + task-shifting + vouchers	9 705 724	327.51	2 646.68
Task-shifting + vouchers	3 201 492	278.06	−372.65

US$: United States dollars.

Notes: The data are from a previously published study^4^ and were used to test the health-adapted superefficiency data envelopment analysis method, producing the policy ranking shown in Table 4. As defined in the original paper, universal public finance refers to making surgery free at the point of care. Task-shifting refers to training non-surgeons to provide a limited bundle of surgical services. Vouchers refer to issuing patients with vouchers for the non-medical costs of care.

**Table 2:** Extended cost–effectiveness analysis of various unrelated preventive and curative health interventions in Ethiopia

Intervention	Government expenditure, US$ × 1 000	Household expenditure averted, US$ × 1 000	No. of deaths averted	No. of cases of impoverishment averted
Rotavirus vaccine	800	180	510	270
Pneumococcal vaccine	1 200	110	1 700	170
Measles vaccine	260	9	890	14
Diarrhoea treatment	50 000	26 000	3 600	40 000
Pneumonia treatment	31 000	15 000	4 100	23 000
Malaria treatment	670	300	410	460
Caesarean section	420	270	590	410
Tuberculosis treatment	6 900	4 400	2 600	6 700
Hypertension treatment	1 300	730	140	1 100

US$: United States dollars.

Notes: The data are from a previously published study^8^ and were used to test the health-adapted superefficiency data envelopment analysis method, producing the policy ranking shown in Table 5.

**Table 3:** Extended cost–effectiveness analysis of various government and nongovernmental interventions for delivery of surgical cancer care in Uganda

Intervention	Cost, US$ per 100 000 population	No. of deaths averted per 100 000 population	No. of cases of impoverishment averted per 100 000 population	No. of cases of catastrophic expense averted per 100 000 population**^a^**	Equity score**^a^**
Universal public finance	3 320	3.0	0.7	4.2	−0.08
Task-shifting	301	3.2	−8.1	−34.8	−0.16
Universal public finance + task-shifting	3 670	8.7	−1.8	−23.1	−0.24
Universal public finance + vouchers	24 470	30.7	123.8	218.6	0.24
Task-shifting + vouchers	13 701	18.7	18.0	57.1	−0.05
Universal public finance + task-shifting + vouchers	25 009	33.6	127.2	218.6	0.23
Two-week mission trip	40 438	1.5	2.4	7.2	0.23
Mobile surgical unit	7 047	42.8	106.6	99.4	0.19
Cancer hospital	54 431	30.3	74.9	81.2	0.13

US$: United States dollars.

^a^ Equity scores were scaled from 1 (most favourable to poorer patients) to −1 (most favourable to richer patients).

Notes: The data were from a previously published study^5^ and were used to test the health-adapted superefficiency data envelopment analysis method, producing the policy ranking shown in Table 6. As defined in the original paper, universal public finance refers to making surgery free at the point of care. Task-shifting refers to training non-surgeons to provide a limited bundle of surgical services. Vouchers refer to issuing patients with vouchers for the nonmedical costs of care. Two-week surgical mission trips and the construction of a cancer hospital are self-explanatory. The modelled mobile surgical unit travelled around Uganda providing surgery at locations not served by a hospital.

Since the purpose of this paper was not the validation of prior analyses, we did not repeat any of these cost‒effectiveness analyses; they were used as examples rather than outcomes of this paper. Similarly, the underlying assumptions in these original papers (for example, that health, financial risk protection and equity may be mutually exclusive) were not tested in this paper. They were, as with all the results used as examples, and were taken at face value.

We compared the results of the new method with two existing methods: traditional cost‒effectiveness analysis (which incorporates only costs and health benefits); and standard data envelopment analysis. Analysis was performed in *R* software, version 3.0 (R Foundation for Statistical Computing, Vienna, Austria). Institutional review board approval was not required, because the analysis used previously published data.

## Results

### Comparing related policies

Table 4 shows the results of applying the three decision-making tools to the analysis of policies to increase access to surgery (Table 1). Traditional cost‒effectiveness analysis would rule out three of the policies (universal public finance, task-shifting plus vouchers for non-medical costs and universal public finance plus vouchers), because they are dominated by other policies, that is, other policies are both less expensive and more effective. Of the remaining policies, a combination of universal public finance plus task-shifting plus vouchers had the least attractive cost‒benefit ratio: over US$ 190 000 per death averted. Standard data envelopment analysis was uninformative: all except one policy (task-shifting plus vouchers) had the maximal value of 1. 

**Table 4:** Comparison of three decision-making tools to determine the value of policy interventions to increase access to surgery

Intervention	Incremental cost–effectiveness ratio^a^	Data envelopment analysis score	Health-adapted superefficiency data envelopment analysis score
Universal public finance	Dominated	1.00	5.84
Task-shifting	Dominated	1.00	1.76
Universal public finance + task-shifting	US$ 1 590 per death averted	1.00	5.38
Universal public finance + vouchers	US$ 53 396 per death averted	1.00	1.98
Task-shifting+ vouchers	US$ 191 515 per death averted	1.00	6.59
Universal public finance + task-shifting + vouchers	Dominated	0.67	0.67

US$: United States dollars.

^a^ A policy is dominated when another policy is both cheaper and more effective.

Notes: We applied the three data analysis methods to a previously published extended cost–effectiveness analysis of various policies to improve access to surgery in Ethiopia (Table 1).^4^ Cost–effectiveness analysis would preclude three policies as dominated. Data envelopment analysis would not give any guidance on how to decide among the six proposed policies. Health-adapted superefficiency data envelopment analysis provides a complete ranking of policies.

By contrast, health-adapted superefficiency data envelopment analysis allowed the policies to be ranked from highest (value score: 6.59) to lowest value (score: 0.67), incorporating both the health and the financial protective effects of these policies. When these effects were included in the decision, the combination of all three policies (universal public finance plus task-shifting plus vouchers) provided the best value for the combination of health and financial risk protection (score: 6.59). The next best policies were universal public finance alone (score: 5.84), which dominated in the cost‒effectiveness analysis, and task-shifting alone (score: 5.38). Task-shifting plus vouchers, which had a lower value score in the traditional data envelopment analysis (score: 0.67) had the same score under health-adapted superefficiency data envelopment analysis (score: 0.67).

### Comparing unrelated policies

Table 5 demonstrates the applicability of the different decision-making tools to the evaluation of multiple, unrelated interventions (Table 2). This is a more realistic scenario than the policies in the first example, which all concerned delivery of surgical services. The second example adds a third output, household expenditures averted, to deaths averted and impoverishment averted. Again, traditional data envelopment analysis was not the most useful tool for decision-making because only three policies scored <  1 and could be ruled out (rotavirus vaccination, malaria treatment and hypertension treatment). Similarly, traditional cost‒effectiveness analysis ruled out five of the nine policies. 

**Table 5:** Comparison of three decision-making tools to determine the value of various unrelated preventive and curative health interventions

Intervention	Incremental cost–effectiveness ratio**^a^**	Data envelopment analysis score	Health-adapted superefficiency data envelopment analysis score
Rotavirus vaccine	Dominated	0.46	0.46
Pneumococcal vaccine	US$ 1 160 per death averted	1.00	2.84
Measles vaccine	US$ 292	1.00	2.43
Diarrhoea treatment	Dominated	1.00	2.36
Pneumonia treatment	US$ 16 067 per death averted	1.00	2.75
Malaria treatment	Dominated	0.70	0.70
Caesarean section	Dominated	1.00	1.51
Tuberculosis treatment	US$ 6 333 per death averted	1.00	1.79
Hypertension treatment	Dominated	0.88	0.88

US$: United States dollars.

^a^ A policy is dominated when another policy is both cheaper and more effective.

Notes: We applied the three data analysis methods to a previously published extended cost–effectiveness analysis of various unrelated preventive and curative interventions in Ethiopia (Table 2).^8^ Data envelopment analysis does not give any guidance on how to decide among the nine proposed policies. Under cost–effectiveness analysis, the policy with the highest health-adapted superefficiency data envelopment analysis value has the worst incremental cost–effectiveness ratio. Health-adapted superefficiency data envelopment analysis provides a complete ranking of policies, even when the policies address different health conditions.

Health-adapted superefficiency data envelopment analysis allowed differentiation among the policies, ranking them from low to high value, and would therefore be more useful than the other analysis tools for prioritizing competing choices. Pneumococcal vaccination had the highest value (score: 2.84) when all outcomes were considered but had the lowest value in traditional cost‒effectiveness analysis (US$ 1160 per death averted). The next best interventions were pneumonia treatment, measles vaccine, diarrhoea treatment and tuberculosis treatment (scores: 1.79‒2.75), followed by caesarean section birth (score: 1.51). 

### Evaluating equity

The data for the third example, an analysis of policies to improve access to surgical cancer care (Table 3), also included two measures of financial risk protection but added a measure of equity. Table 6 shows that cost‒effectiveness rules out all but two policies (task-shifting and the mobile surgical unit). Traditional data envelopment analysis was again unhelpful for decision-making; only one policy (universal public finance for surgery plus task-shifting) scored <  1.

**Table 6:** Comparison of three decision-making tools to determine the value of various government and nongovernmental interventions for improving the delivery of surgical oncology services, when equity is added

Intervention	Incremental cost–effectiveness ratio**^a^**	Data envelopment analysis score	Health-adapted superefficiency data envelopment analysis score
Universal public finance	Dominated	1.00	2.12
Task-shifting	US$ 94 per death averted	1.00	11.07
Universal public finance + task-shifting	Dominated	0.89	0.89
Universal public finance + vouchers	Dominated	1.00	2.08
Task-shifting+ vouchers	Dominated	1.00	1.00
Universal public finance + task-shifting + vouchers	Dominated	1.00	2.05
Two-week mission trip	Dominated	1.00	1.00
Mobile surgical unit	US$ 99 per death averted	1.00	4.82
Cancer hospital	Dominated	1.00	1.00

US$: United States dollars.

^a^ A policy is dominated when another policy is both cheaper and more effective.

Notes: We applied the three data analysis methods to a previously published cost–effectiveness analysis of government policies and nongovernmental platforms for improving the delivery of surgical oncology services in Uganda (Table 3).^5^ Data envelopment analysis does not give any guidance on how to decide among the six proposed policies. Cost–effectiveness analysis and health-adapted superefficiency data envelopment analysis favour similar policies. Health-adapted superefficiency data envelopment analysis, however, offers a ranking of policies that appear dominated by cost–effectiveness analysis alone.

Health-adapted superefficiency data envelopment analysis also ranked task-shifting (value score: 11.0) and mobile surgical units (score: 4.82) the highest, but in addition produced a clear ranking among all policies, including the dominated ones. With equity added to the equation, a decision-maker using health-adapted superefficiency data envelopment analysis would be guided towards task-shifting, given the results of the underlying extended cost‒effectiveness analysis. If this were not feasible, or in the interim while it was being scaled up, mobile surgical units might be the best choice to deliver surgical oncology care.

## Discussion

In this paper, we developed and tested a method for decision-making in health policy when the population’s preferences among potential outcomes are unknown. We found that health-policy-adapted superefficiency data envelopment analysis was capable of incorporating multiple attributes and functioned better than incremental cost‒effectiveness ratios or traditional data envelopment analysis in health-policy settings.

Since cost‒effectiveness analysis relies on a ratio of incremental costs over incremental health effects (measured often as disability-adjusted life-years, quality-adjusted life-years or absolute numbers of lives saved), it ignores the non-health effects of policies. As such, this common decision-making method does not fully represent the wishes of a population, a weakness that has led to counterintuitive results^17^ and, in some cases, an implicit prohibition against using ratios for decision-making altogether.^18^

In moving towards UHC, we need to look at the effects of health policies on multiple domains, including, for example, health, financial well-being and equity. The assumptions made in traditional cost‒effectiveness analysis become unsound. As this paper shows, the multi-attribute value of policy proposals is often categorically different from their cost‒effectiveness. Policies that are dominated under cost‒effectiveness analysis assumptions, and therefore declared unworthy of further study, become efficient, and sometimes the most efficient, with multi-attribute decision-making. Our method produces a rank-ordering of policies, allowing more comprehensive decisions to be made.

Given that between 20% and 40% of health spending globally is wasted because of inefficiency,^19^ health-adapted superefficiency data envelopment analysis can provide valuable information to increase efficiency. Data envelopment analysis has been used to evaluate health-care delivery by facilities in various low- and middle-income countries^20^^–^^24^ and management of chronic disease in American states,^25^ and even as a way to evaluate the relative merit of scientific research projects.^26^ Our new method, however, allows data envelopment analysis to be applied to policies and to be modified for health-specific contexts.

Our new method has its limitations. Value, as defined by this method, can only be a proxy for decision-making. The method does not avoid the need for formal evaluations of population preferences over health improvement, financial risk protection and equity. These evaluations are difficult to perform, however, and this new method of analysis allows health policy choices to be made in the absence of quantitative evidence on patient preferences. In addition, only quantitative inputs and outputs can be considered in this new method. Non-quantitative factors which may be important to a policy-maker, such as political will, must either be quantified or be excluded from the analysis. Finally, it is not a method for policy evaluation, but for decision-making after evaluation. Cost‒effectiveness analyses, extended and otherwise, can be employed to predict the outcomes of potential policies, but cannot by themselves guide the policy-maker in how to choose given these outcomes. We developed this new method to move from evaluation to decision-making. Since no single score can dictate policymak*i*ng, our method can be used to help guide a policy-maker as to the relative value of a proposed policy. Other, competing priorities and political realities must be balanced with these results.

Despite these weaknesses, the proposed new method has many strengths for health policy decision-making. First, it allows a holistic, multidimensional evaluation of health policies. As health policies assessments begin to incorporate all three aspects of the UHC framework,^9^ the method will permit multi-attribute decision-making that can incorporate any quantifiable attribute or determinant of health. The method is not limited to health, equity and financial risk protection, as in the examples presented here.

Second, the analysis is relatively easy to perform. We used *R*, a free and publicly available statistical software, but other software programs include add-in modules for data envelopment analysis. To facilitate use of our method, we have developed a stand-alone, free, web-based module (available at: http://markshrime.com/research-tools/).

Finally, the new method does not require a judgement about the relative importance of each policy domain. Population-level preference studies are needed to quantify the how much a country’s population values health protection, cost, equity and financial risk protection. Until then, our method of analysis offers guidance for policy-makers.

In conclusion, health-adapted superefficiency data envelopment analysis is an adaptable tool for decision-making in the sustainable development era. The method is a formalization of the value model in health, flexibly incorporating comprehensive factors within both outcome and input domains. As such, the method can be used in place of cost‒effectiveness analysis and other ratio methods for decision-making. This paper demonstrates that this method is not only feasible, but by providing a rank order of policies, more aptly represents the multidimensional decision-making that faces policy-makers daily.
